# A guide to measuring expert performance in forensic pattern matching

**DOI:** 10.3758/s13428-024-02354-y

**Published:** 2024-03-14

**Authors:** Samuel G. Robson, Rachel A. Searston, Matthew B. Thompson, Jason M. Tangen

**Affiliations:** 1 School of Psychology, The University of Queensland, St Lucia, QLD Australia; 2 School of Psychology, The University of New South Wales, Kensington, NSW Australia; 3 School of Psychology, The University of Adelaide, Adelaide, SA Australia; 4 School of Psychology, Murdoch University, Murdoch, WA Australia; 5 Centre for Biosecurity and One Health, Harry Butler Institute, Murdoch University, Murdoch, WA Australia

**Keywords:** Forensic science, Decision-making, Expertise, Signal detection, Fingerprints, Proficiency tests, Forensic pattern matching

## Abstract

Decisions in forensic science are often binary. A firearms expert must decide whether a bullet was fired from a particular gun or not. A face comparison expert must decide whether a photograph matches a suspect or not. A fingerprint examiner must decide whether a crime scene fingerprint belongs to a suspect or not. Researchers who study these decisions have therefore quantified expert performance using measurement models derived largely from signal detection theory. Here we demonstrate that the design and measurement choices researchers make can have a dramatic effect on the conclusions drawn about the performance of forensic examiners. We introduce several performance models – proportion correct, diagnosticity ratio, and parametric and non-parametric signal detection measures – and apply them to forensic decisions. We use data from expert and novice fingerprint comparison decisions along with a resampling method to demonstrate how experimental results can change as a function of the task, case materials, and measurement model chosen. We also graphically show how response bias, prevalence, inconclusive responses, floor and ceiling effects, case sampling, and number of trials might affect one’s interpretation of expert performance in forensics. Finally, we discuss several considerations for experimental and diagnostic accuracy studies: (1) include an equal number of same-source and different-source trials; (2) record inconclusive responses separately from forced choices; (3) include a control comparison group; (4) counterbalance or randomly sample trials for each participant; and (5) present as many trials to participants as is practical.

Forensic examiners observe crime scene trace evidence – such as ballistic impressions, bitemarks, toolmarks, shoe prints, tire tracks, faces in CCTV footage, handwriting, and fingerprints – to determine the source of the trace. When examiners get these decisions right, their opinions can help law enforcement and the courts to convict the guilty and exonerate the innocent. When examiners get these decisions wrong, however, their evidence may contribute to wrongful convictions (Garrett & Neufeld, 2009) or failures to identify key suspects in criminal investigations. Landmark reports from the National Research Council (2009), the President’s Council of Advisors on Science and Technology (2016), and the American Association for the Advancement of Science (2017) have put a spotlight on such errors, but there is disagreement about how best to quantify them (Albright, 2021; 2022; Dror, 2020; Dror & Rosenthal, 2008; Koehler, 2013, 2017). Here we offer a descriptive guide to measuring human performance in forensic pattern matching disciplines using the framework of signal detection theory.

This guide is intended for cognitive scientists and applied researchers who are interested in measuring human decision making in forensic pattern matching disciplines, for forensic scientists who are interested in understanding how and why scientists measure performance in ways that may deviate from decision-making frameworks used in practice, and for others in the legal system interesting in making sense of scientific studies on human performance in forensic science. We begin this guide by introducing signal detection theory and explaining how it can be applied in the specific domain of fingerprint identification. We then introduce several measurement models commonly used by cognitive scientists to quantify human performance: proportion correct, sensitivity, specificity, diagnosticity ratio, d prime (dʹ), A-prime (Aʹ) and empirical area under the curve (AUC). Following this, we use a resampling method to explore a range of common scenarios that arise when measuring performance in forensic pattern-matching domains and examine how these scenarios might affect measurement.

Of course, we are not the first to explore signal detection theory and we do not intend for this to be a comprehensive introduction to the topic. There are many texts that provide in-depth background to the framework, and we encourage interested readers to seek these out (e.g., Macmillan & Creelman, 2005). This paper is rather a demonstration of how signal detection models can be applied to real-world data and decisions, such as those made by forensic experts like fingerprint examiners. Within this context, we show that some models can drastically distort performance in certain scenarios and therefore the conclusions that are drawn. While this guide is intended to be more descriptive than prescriptive, we also offer practical considerations to some of the measurement problems arising within each scenario. These considerations can help guide the design and critical evaluation of studies of human performance in forensic pattern matching and other contexts where signal detection models are applied to real-world human performance data.

## Signal detection in forensic science

People are frequently required to choose between two options when navigating the world: Is that person a threat or not? Am I pregnant or not? Is there a fire or not? These are binary decisions; the answer is either yes or no, and one’s judgment is either true or false. Many decisions in forensic pattern matching are also dichotomous. For instance, a firearms expert must determine if a bullet was fired from a particular gun or not. An expert in facial comparison must determine whether a photograph matches a suspect or not. A fingerprint examiner must determine whether a fingerprint was left by a suspect or not. Forensic examiners may at times make non-binary judgments. For example, when analyzing blood spatter, an examiner may need to determine what type of weapon was used, where it made contact, and from what direction. These kinds of non-binary determinations are beyond the scope of this paper. Quantifying performance for binary decisions, however, is central to research on expert decision-making in forensic science: Are experts better than novices? How does procedure A compare to procedure B? How well is this examiner performing (Smith & Thompson, 2019)? Signal detection is helpful for answering these questions.

There is a long history of using signal detection theory to evaluate performance (see Wixted, 2020). The core ideas were first encapsulated in 1860 (Fechner, 1860/1966) and formally theorized in the mid-20th century (Marcum, 1947; Peterson et al., 1954). Since then, it has been adopted by researchers in many different fields, including diagnostic medicine, weather forecasting, eyewitness identification, and personnel selection (Tanner & Swets, 1954; Green & Swets, 1966; Swets, 1973; Swets, 1988; Swets et al, 1961; Wixted & Mickes, 2018). Phillips et al. (2001), to our knowledge, first proposed that signal detection theory be applied as a method for evaluating performance in the forensic sciences.

According to signal detection theory, performance is characterized by how well a system (an individual, group, technique, or department) can distinguish between what it seeks to find (the signal) against what it seeks to filter out (the noise). The ability to tell signal from noise is known as *discriminability*^1^*.* In radar operation, for instance, the signal consists of dots on a screen that represent enemy warships, whereas the noise consists of dots on a screen that represent everything else. In forensic pattern-matching, an examiner uses their physical senses to judge the degree of similarity between trace evidence and a reference sample (Albright, 2021). They must decide whether the evidence originated from the same source or not, but there is also a correct answer to this question; the evidence either originated from the source in reality or it did not. Thus, signal is when the trace evidence and the reference sample come from the same source. Noise is when the evidence and the reference sample are not from the same source.

To measure how well a person can discriminate between signal and noise, their judgments can be compared with the ground truth. Although no one can know the truth of trace evidence in casework for certain, researchers can develop materials that have been obtained in a way that identifies the source. Controlled experiments allow researchers to gather data on how well people’s judgements align with ground truth. These data could be used for the scientific study of human performance, and for operational purposes such as diagnostic accuracy studies, proficiency tests, system-level black box studies, or ongoing management and quality assurance. With this information, signal detection theory then offers a way of quantifying how well observers can detect whether trace evidence came from the same source as a reference sample.

A key benefit of a signal detection approach to measuring human performance is that it allows one to distinguish between accuracy and response bias (Macmillan & Creelman, 1990, 2005). Accuracy refers to the number of correct decisions whereas response bias refers to favoring one outcome over another, such as saying ‘signal’ more often than ‘noise’. Imagine that 1% of the population has a particular disease. It is possible for a doctor to be 99% accurate in their diagnoses simply by saying that every patient does not have the disease. Likewise, if this doctor wanted to detect every person with the disease, they could simply say that every patient has the disease in which case they would correctly detect the disease 100% of the time. These are examples of extreme response bias, yet in both instances there is no indication that the doctor can effectively diagnose the disease; they have not demonstrated that they can discriminate signal from noise (the presence vs. absence of the disease). Accuracy is confounded by response bias. Using signal detection theory and discriminability resolves this issue. We return to this idea later.

Several studies of forensic performance have employed signal detection theory in their analyses (e.g., Busey et al., 2022; Carter et al., 2020; Growns & Kukucka, 2021; Searston et al., 2016; Tangen et al. 2011; Thompson et al., 2013). More recently, Arkes and Koehler (2022), and Smith and Neal (2021), have also called for widespread adoption of signal detection theory in cognitive forensic research. However, even if many cognitive and forensic researchers do adopt this approach, the models, materials, and study design that they adopt will differ. To understand and communicate the value of forensic decisions, we need reproducible methods and robust models. In the next section, we run through how to measure performance with signal detection theory using a recent experiment that we conducted with fingerprint experts as an example.

## Signal detection in fingerprint identification

Police departments employ fingerprint examiners to identify the source of fingerprints discovered at crime scenes. In this situation, the question is whether this fingerprint came from a specific suspect's finger or not. Throughout their careers, fingerprint examiners spend thousands of hours comparing and inspecting highly structured prints, and then present their findings to factfinders in criminal and civil cases. Prior research has shown that fingerprint examiners outperform novices on a range of perceptual and cognitive fingerprint tasks (Robson et al., 2021; Searston et al. 2016; Searston & Tangen, 2017a, 2017b; Tangen et al., 2011; Thompson et al., 2014; Thompson & Tangen, 2014). Here, we report the results of an original experiment comparing the latent fingerprint matching performance of qualified, court practicing fingerprint experts to untrained novices using a Signal detection framework.

We conducted an experiment to investigate differences in fingerprint comparison performance between professional examiners and novices (preregistered project: https://osf.io/h4tjq/wiki/home). Participants were 44 fingerprint experts and 44 age, gender, and education-matched novices. We presented each participant with 24 pairs of prints collected from actual case files that were selected as being very difficult to distinguish. We also knew the ground truth of each fingerprint pair, i.e., whether or not they matched. Moreover, each yoked expert-novice pair was presented with a unique set of 12 same-source prints and 12 different-source prints that were randomly sampled from a larger pool of 48 fingerprint pairs.

**Fig. 1:**
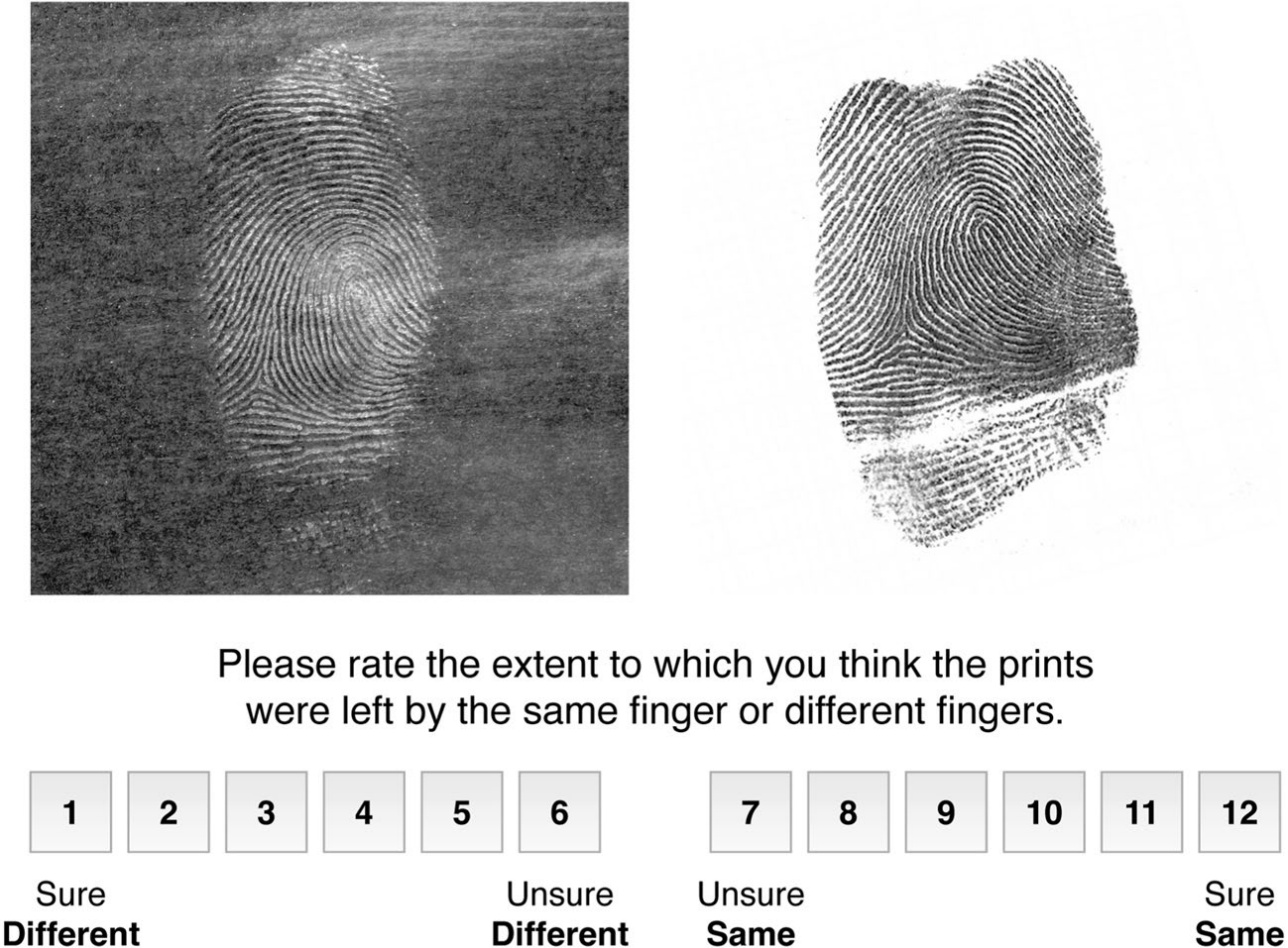
An example trial from a fingerprint matching experiment. On each trial, participants were shown a crime-scene print on the left and a candidate print on the right. They were asked to rate their confidence from 1 (sure different) to 12 (sure same). In some cases, the two prints were from the same person; in others, the prints were similar but originated from two different people

On a 12-point scale ranging from 1: sure different to 12: sure same, we asked participants to rate the extent to which they thought the prints came from the same finger or different fingers. Every rating from 1 to 6 was therefore coded as a different-source judgment and ratings from 7 to 12 were coded as a same-source judgment. Participants did not receive feedback on any trials. The task is illustrated in Fig. 1. Note that we are not necessarily recommending that forensic casework decisions be made using a confidence rating such as this. These scales are, however, useful for cognitive scientists and applied researchers interested in measuring human perceptual performance, and how different groups or examiners may differ from one another in their abilities.

**Fig. 2:**
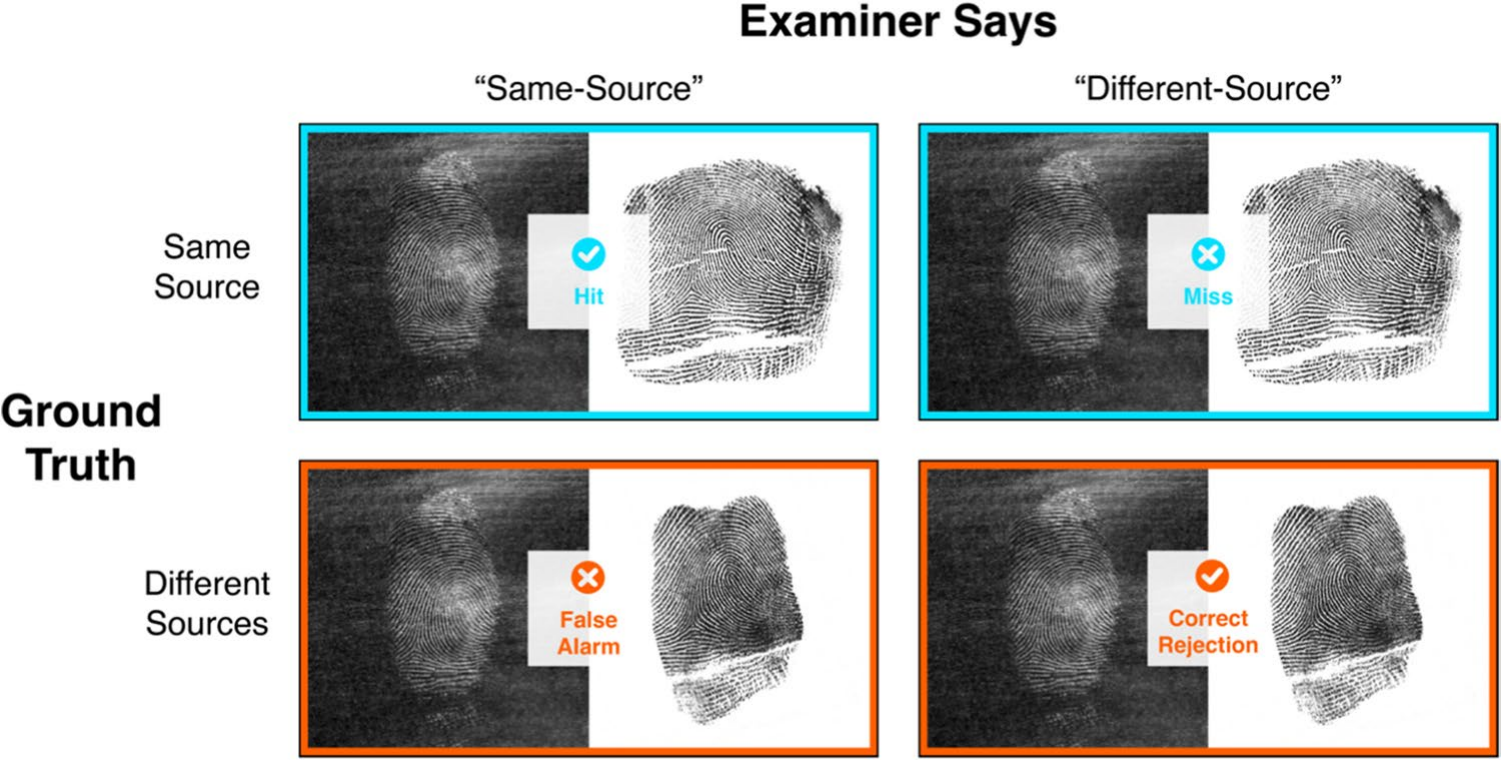
When comparing fingerprints, there are four possible outcomes. When an examiner decides whether two prints came from the same source or not, their decision is compared to the ground truth. If two prints originated from the same source and the examiner says “same”, the outcome is a hit, but if they say “different”, the outcome is a miss. In contrast, if two prints originated from different sources and a person says “different”, the outcome is a correct rejection, but if they say “same”, the outcome is a false alarm. If one uses a 12-point rating scale, decisions can be collapsed such that ratings from 1 to 6 are coded as a different-source judgment and ratings from 7 to 12 are coded as a same-source judgment

To establish how well the fingerprint examiners and novices performed on our latent fingerprint matching task, we can use a signal detection framework. There are four possible choice outcomes when each judgment is matched against the ground truth (see Fig. 2). An examiner can correctly say that two fingerprints from the same source are the “same” (a hit), or incorrectly say they are “different” (miss); the examiner can also correctly say that two prints from different sources are “different” (correct rejection), or incorrectly say they are the “same” (false alarm). Performance may be judged in a variety of ways by adjusting how we tally up the number of decisions that fall into each of these categories.

**Fig. 3:**
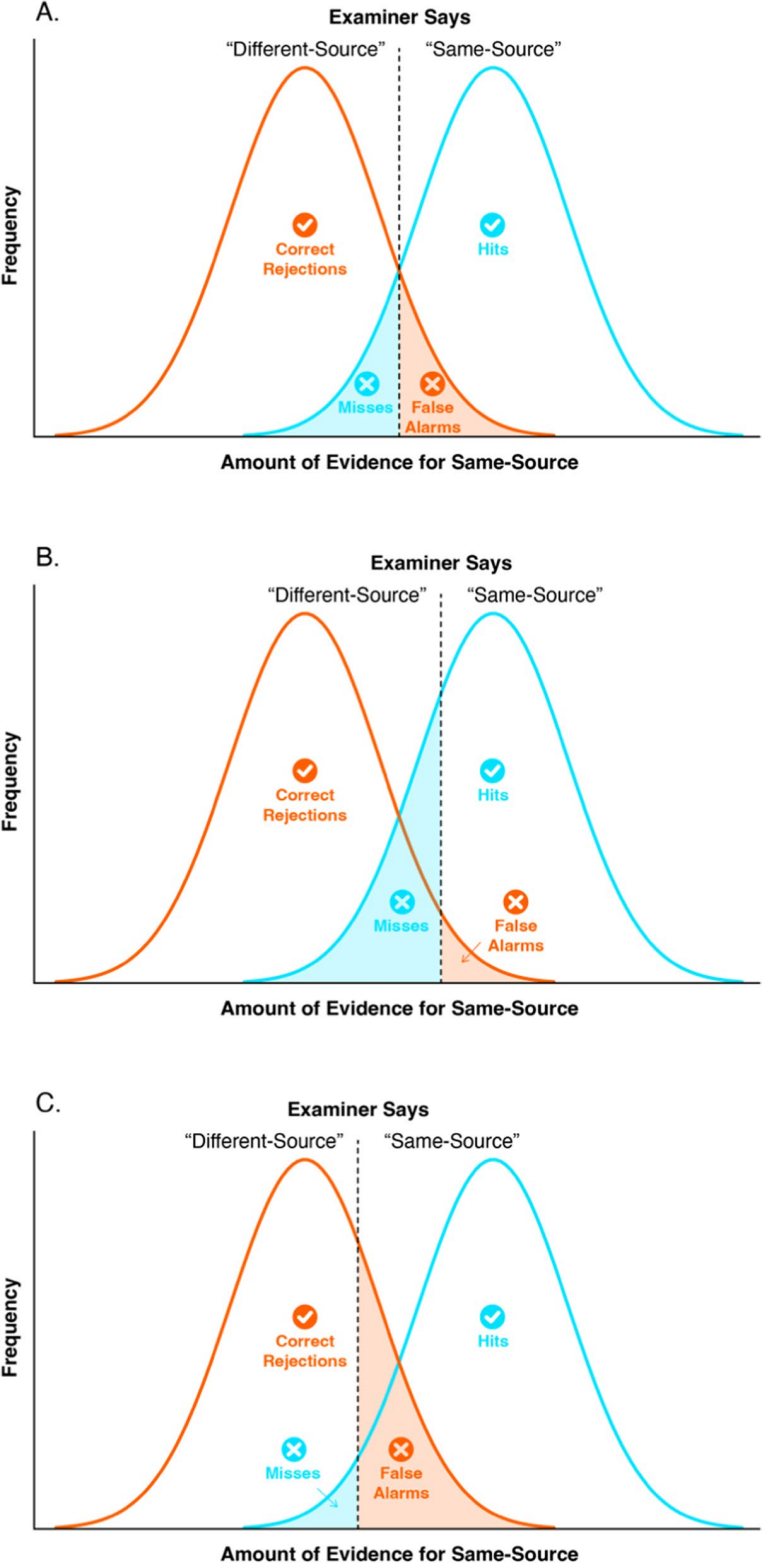
A representation of the relative proportion of hits, misses, false alarms, and correct rejections for signal and noise distributions that overlap. The *vertical line* in each panel depicts the decision threshold or response bias. In **A**, the system has no response bias. In **B**, the system has a conservative response bias and so there are more correct rejections and misses. In **C**, the response bias is liberal, resulting in a greater number of hits and false alarms

A person whose judgments consist of only hits and correct rejections performs perfectly, whereas a poor performer frequently makes misses and false alarms. However, telling fingerprints apart, as with most real-world decisions, is not always clear cut. In a set of fingerprints with a large amount of variation, sometimes two prints deposited by the same finger can look very different from one another, and sometimes two prints from different fingers can appear very similar. Same-source pairs and different-source pairs can be confused. This ambiguity can be represented probabilistically via two overlapping signal and noise distributions (see Fig. 3). The less an examiner confuses signal for noise, and noise for signal, the less overlap there is between these distributions, and the better the examiner’s performance.

Additionally, a person’s response bias, or criteria for making decisions, can range from highly liberal (errs on the side of saying same-source) to highly conservative (errs on the side of saying different-source). A more conservative response bias produces more correct rejections, but also more misses. A more liberal criterion produces more hits, but also more false alarms. The overlap between signal and noise, and the response bias, determine the proportion of hits, misses, correct rejections, and false alarms of a human observer. In practice, the response bias (or decision threshold) that examiners adopt is consequential. For example, Thompson (2023) has demonstrated, using a Signal Detection model, that even small shifts in response bias can dramatically impact the likelihood of conviction or acquittal.

We can classify the decisions from the earlier fingerprint task into hits, correct rejections, misses, and false alarms for experts (Table 1) and novices (Table 2)^2^. Values in Tables 1 and 2 show that both experts and novices performed better than chance, but both groups made mistakes on occasion. Experts generally fared better than novices, making fewer mistakes overall in terms of both misses and false alarms.

**Table 1:** Binary classification table for expert participants

		Expert says			
		“Same”	“Different”	Row total	
Ground truth	Same source (targets)	396 (Hits/True positives)	119 (Misses/False negatives)	515	Sensitivity = 76.89% (396/515)
	Different source (distractors)	64 (False alarms/false positives)	477 (Correct rejections/true negatives)	541	Specificity = 88.17% (477/541)
	Column total	460	596	1056	
		PPV = 86.09% (396/460)	NPV = 80.03% (477/596)		Proportion correct = 82.67% (873/1056)

**Table 2:** Binary classification table for novice participants

		Novice says			
		“Same”	“Different”	Row total	
Ground truth	Same source (targets)	362 (Hits/true positives)	153 (Misses/false negatives)	515	Sensitivity = 70.29% (362/515)
	Different source (distractors)	282 (False alarms/false positives)	259 (Correct rejections/ true negatives)	541	Specificity = 47.87% (259/541)
	Column total	644	412	1056	
		PPV = 56.21% (362/644)	NPV = 62.86% (259/412)		Proportion correct = 58.81% (621/1056)

Delving deeper into Tables 1 and 2, we can express performance with several diagnostic accuracies: Positive predictive value (PPV) is the likelihood that the fingerprints came from the same source when responded “same.” In our study, the PPV for the experts was 86%. The negative predictive value (NPV), on the other hand, is the likelihood that the fingerprints came from different sources when responded “different.” In our study, the NPV for the experts was 80%. Predictive values can be useful to factfinders because they express the *reliability* of the decision made by a forensic practitioner (Mickes, 2015; Smith & Neal, 2021). However, predictive values in Table 1 do not necessarily reflect the operational decision-making ability of examiners because the task was completed under time constraints and without the usual tools that fingerprint examiners have at their disposal, and outside a broader system. Moreover, for the purposes of determining human performance, sensitivity and specificity are more relevant. Unlike PPV and NPV, sensitivity and specificity are conditioned on ground truth rather than the examiner’s judgments, and hence describe their *validity*.

Together, sensitivity and specificity express how well a person or group can distinguish signal from noise. Sensitivity indicates how likely it is that a person will say “same source” when the fingerprints actually come from the same person. Specificity indicates how likely it is that a person will say “different source” when two fingerprints come from different people. When sensitivity is higher than specificity, the response bias is relatively liberal, whereas response bias is conservative when specificity is higher than sensitivity.

Tables 1 and 2 indicate the relatively comparable sensitivity of experts (77%) and novices (70%), but specificity is higher for experts (88%) than for novices (48%). Expert examiners clearly perform better than novices overall, but examiners are also more careful than novices to declare that two fingerprints are from the same source. Sensitivity and specificity each provide only a partial picture of performance. Consider a scenario in which experts had higher specificity than novices, but lower sensitivity. It would be unclear which group is better. Frequently, researchers will want to distil performance into a single value to unambiguously compare individuals or groups.

## Common measurement models of performance

**Fig. 4:**
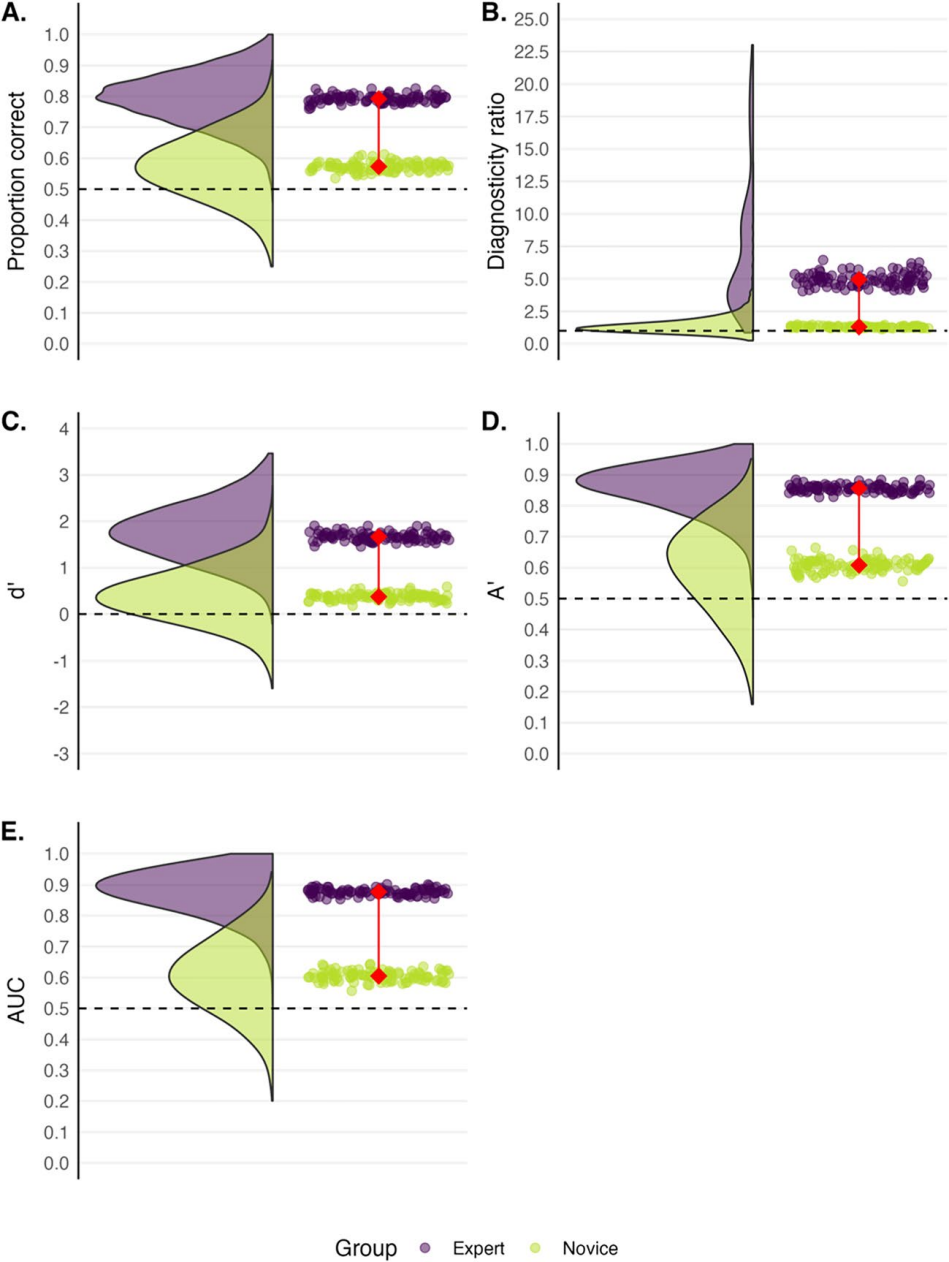
A representation of resampled expert (*purple*) and novice (*green*) data for proportion correct (**A**), diagnosticity ratio (**B**), dʹ (**C**), Aʹ (**D**) and AUC (**E**). The *rainclouds* depict the distributions of every participant’s score across 100 expert and novice samples. Each *raindrop* indicates a group mean score and the connected *red diamonds* represent the average difference of the group means. *Dashed lines* represent chance performance

The aim of this section is to introduce some commonly used single-value models of human performance. Rather than simply presenting data from the comparison task above to discuss these models, we used a resampling method to generate more stable estimates of performance and come to a better sense of the performance variation. We first obtained the means and standard deviations for the confidence ratings of the experts and novices for the same-source and different-source trials in the fingerprint matching task described earlier. We then randomly sampled datapoints from beta distributions based on these means and standard deviations. Specifically, we sampled confidence ratings for 12 same-source and 12 different-source trials for 44 hypothetical experts and 44 hypothetical novices 100 times over as if we had conducted the study many times. In Fig. 4, we present the data for proportion correct, diagnosticity ratio, dʹ, Aʹ, empirical AUC. The rainclouds represent the distribution of scores across the 100 resampled experiments.

### Proportion correct

Perhaps the most used measure of performance is proportion correct (or percent correct), which is a tally of the total number of hits and correct rejections divided by the total number of trials. Several forensic expertise studies have used proportion correct as a measure of ability (Bird et al., 2010; Searston & Tangen, 2017a; Tangen et al., 2011; Thompson & Tangen, 2014; White & Dunn et al., 2015). Proportion correct provides an intuitive sense of performance by showing how many times a person answered correctly. As seen in Tables 1 and 2, experts (83%) have a higher proportion correct than novices (59%) on the fingerprint discrimination task. Experts were incorrect on 17% of trials whereas novices were incorrect on 41% of the trials. Figure 4A also shows the distribution for proportion correct across the resampled expert (*M* = 79%) and novice (*M* = 57%) data.

### Diagnosticity ratio

Diagnosticity ratio is a model of performance that combines sensitivity and specificity into one value. More specifically, it is a ratio of the odds of a same-source decision on same-source trials relative to the odds of a same-source decision on different-source trials. To compute the ratio, sensitivity is divided by the inverse of the specificity (i.e., hit rate divided by the false alarm rate). Diagnosticity ratios have been used in several forensic studies, most notably in comparisons between sequential and simultaneous eyewitness lineups (see Wells & Lindsay, 1985; Steblay et al., 2011).

Taking the data from Tables 1 and 2 reveals that the diagnosticity ratio of experts in the fingerprint matching task was 6.50, and for novices it was 1.35. Experts clearly performed better than the novices according to these ratios. Figure 4B, however, shows a tendency for these odds ratios to take on extreme values, especially if performance is not collapsed across participants. Extreme values occur when the false alarm rate or hit rate approach zero or one. Mickes et al. (2014) and Wixted and Mickes (2018) have also articulated why a diagnosticity ratio is frequently a poor measure of performance for a variety of other reasons, including the influence of response bias. Moreover, the diagnosticity ratio is more closely related to PPV than to discriminability (Wixted & Mickes, 2018). For these reasons, we do not see much utility in using a diagnosticity ratio to gauge forensic performance, and so we do not discuss it further.

### dʹ (d-prime)

Performance in signal detection theory is conceptualized as two overlapping Gaussian distributions, one representing signal and the other noise. In any task, however, a decision-maker uses some sort of threshold or criteria to make a decision. In the fingerprint discrimination task, for example, experts erred on the side of caution, saying “different” more frequently than “same”, whereas novices said “same” more frequently than “different”. If an observer shifts their response bias, however, this can alter the number of hits, correct rejections, false alarms, and correct rejections (see Fig. 3). By extension, the values for proportion correct and diagnosticity change as well. Signal detection models such as dʹ (d-prime), Aʹ, and empirical AUC, have been devised to account for differences in response bias.

One of the most widely used signal detection measures is dʹ (Green & Swets, 1966). It is a measure of the distance between the signal and noise distributions, such as those depicted in Fig. 3. If the mean of the signal distribution is one standard deviation away from the mean of the noise distribution, then dʹ is equal to one. A higher dʹ means that the distance between the two distributions is greater, indicating that the observer can better distinguish noise and signal. To calculate dʹ, the standardized false alarm rate is subtracted from the standardized hit rate (Macmillan & Creelman, 2005). By standardizing these values, the model factors in response bias by assuming that the values fall on hypothetical normal distributions. The variances of these distributions are also assumed to be homogeneous. In fact, dʹ monotonically maps onto proportion correct when there is an equal ratio of noise and signal trials (i.e., match and no-match trials) and there is no response bias. dʹ has been used as the key measurement model in several forensic matching studies (e.g., Estudillo et al., 2021; Towler et al., 2017; Vogelsang et al., 2017).

A common issue with using dʹ is that the performance estimates are unbounded, potentially being infinite or undefined when the hit rate or false alarm rate are equal to zero or one. There are a few ways of addressing this issue. One solution is to aggregate data from several participants. In doing so, the chance of obtaining a value of zero or one is reduced since a larger sample is used to calculate the hit and false alarm rates. However, this solution is not viable if a researcher is interested in the performance of each individual, nor does it guarantee that the values will be appropriate after aggregation.

At least two computational solutions are also possible. Before computing dʹ (or even the diagnosticity ratio), a correction can be made. One option is Macmillan and Kaplan’s (1985) recommendation of converting all values of zero to 0.5/*n* and all rates of one to (*n* – 0.5)/*n*, where n is equal to the number of signal or noise trials. Another option is the log-linear method (Hautus, 1995) where 0.5 is added to the number of hits and false alarms and 1 is added to the total number of signal and noise trials. For further discussion of the costs and benefits of these methods see Stanislaw & Todorov (1999). In this paper, we adopted Macmillan and Kaplan’s (1985) correction method. Figure 4C shows the distribution for dʹ across the resampled expert and novice data.

### Aʹ (A-prime)

Aʹ (A-prime; Pollack & Norman, 1964) has been proposed as a non-parametric alternative to dʹ. However, the assumption that it is non-parametric has been challenged (Macmillan & Creelman, 1996; Verde et al., 2006; Pastore et al., 2003). Aʹ is nevertheless often used when the signal and noise distributions are presumed to have unequal variance. Rather than modelling the distance between the signal and noise distributions, Aʹ uses a ROC function to model performance. ROC analyses have long been relied on to measure discriminability in medicine (Lusted, 1971, Metz, 1978; Pepe, 2000) and some have suggested they be used for evaluating forensic decision-making (Gronlund et al., 2014; Mickes et al., 2012). Few forensic pattern matching studies use Aʹ, but it is popular in more basic categorization research (see Zhang & Mueller, 2005).

A ROC curve is a two-dimensional plot of the hit rate (*y*-axis) and false alarm rate (*x*-axis). Aʹ is calculated using only a single hit rate and false alarm rate, which define a single point on the two-dimensional plane. A series of quasi-ROC curves (lines) can pass through this point, each with a different gradient, which extrapolate how the hit rate and false alarm rate might change with response bias. The area underneath each of these lines is a polygon with a certain area. Aʹ is defined as “the average of the maximum area and minimum area under the proper ROC curve constrained by the hits and false alarms.” The greater the area beneath a ROC curve, the better the performance. If the hit rate is one and the false alarm rate is zero, the area underneath the ROC curve would fill up the entire plane and Aʹ is equal to one. Performance is perfect. In signal detection terms, there is no overlap between signal and noise. For a person performing at chance levels, the ROC curve would be a straight line running from the bottom left to the top right of the plane with an area underneath of .5. An area of .5 is equivalent to signal and noise distributions that overlap completely.

Zhang and Mueller (2005) made a correction to the original computation of Aʹ. This correction computes the area differently depending on where the false alarm rate and hit rate intersect. We use this corrected method here. In instances where the false alarm rate was greater than the hit rate, we used the inverse of both values and subtracted the output from one. Figure 4D shows the distribution for Aʹ across the resampled expert and novice data.

### Empirical AUC

Aʹ and dʹ are examples of theoretical discriminability because they are not solely based on empirical data. Performance at one decision threshold is extrapolated to others by making assumptions. Using theoretical estimates of performance allows one to account for changing response biases given only a single hit rate and false alarm rate. However, these assumptions can be erroneous. The empirical area under the curve (AUC) is a signal detection model of performance that does not rely on underlying assumptions about signal and noise.

Like Aʹ, empirical AUC is based on a ROC curve, but its computation requires a hit rate and false alarm rate at *multiple* decision thresholds rather than just one. Recall that the participants in our fingerprint task from earlier provided a response about whether two prints came from the same source using a 12-point scale (1 = “sure different”, 12 = “sure same”). This response scale allows us to compute a hit rate and false alarm rate at 12 different points, forming a curve when plotted on a two-dimensional plane. The area under a ROC curve represents performance; an area of one indicates perfect performance whereas a value of .5 indicates chance performance. Empirical AUC can be computed in several ways, but we used the pROC package (Robin et al., 2011) in R, which uses the trapezoidal rule. Conceptually, this method involves adding together the area of several trapezoids using the points along the ROC curve. Figure 4E shows the distribution for empirical AUC across the resampled expert and novice data.

Several forensic performance studies have used empirical AUC as a key measurement model (Mickes et al., 2012; White & Phillips et al., 2015; Wixted & Mickes, 2018). Models of discriminability such as dʹ and Aʹ are useful when one knows the underlying distribution of signal and noise. However, for real-world decisions, some scholars advocate for atheoretical models like empirical AUC (Wixted & Mickes, 2018) because no assumptions need be made about how signal and noise vary in reality.

### Summary and considerations

There are several factors that researchers might consider when deciding which performance model to use. If underlying latent variables are of interest, then dʹ and Aʹ may be preferable whereas an empirical measure like AUC may be more suitable when interested in real-world performance. If a parametric measure is unsuitable, then Aʹ and AUC may be preferable (whether Aʹ is truly non-parametric, however, has been questioned). As continuous measures of similarity (e.g., 1 to 12) are more sensitive than dichotomous measures (e.g., same-source/different-source), empirical AUC may be preferred over dʹ or Aʹ. That said, a confidence rating scale may not reflect the options available to examiners in routine casework. The underlying assumptions of each model can have important implications for how researchers measure performance and the conclusions they might draw (for discussion, see Brady et al., 2023). There are also other performance models that we have not explored here (see, for example, Macmillan & Creelman, 1990; Rotello et al., 2008; Verde et al., 2006).

Whatever decision a researcher makes with respect to these models, the reasons for that decision, as well as how the model was computed and corrected, should be made explicit. For example, were the data aggregated among participants? Were extreme values corrected? How was the AUC calculated? We generally recommend that all analytic decisions and data be made as transparent as possible so that researchers can better understand and replicate each other’s work. These choices should also ideally be made prior to viewing and analyzing the data to reduce selective reporting of results (Chin et al., 2019), a practice known as preregistration (see also the case for Registered Reports outlined by Chin et al, 2020). For the remainder of this paper, we explore how various performance models are affected by participant response patterns and experimental design variables.

## Resampling method

For the remainder of the paper, we explore a variety of scenarios researchers may frequently encounter. We use a data resampling method to graphically demonstrate how performance models (e.g., proportion correct, dʹ, Aʹ and AUC) are affected by variables such as response bias, prevalence, inconclusive responses, ceiling effects, case sampling, and number of trials. We will discuss how these variables can affect interpretations about expert performance in forensics. Knowing the kinds of situations that can have a significant impact on performance can help researchers make more informed decisions about which models to use and how to avoid drawing inaccurate conclusions from their observations.

The general methodology was quite similar in each section. It extends on the data resampling method described earlier. We obtained the means and standard deviations for the expert and novice confidence ratings in the fingerprint discrimination task introduced above. We used these values to define distributions from which we then randomly sample data points (confidence ratings) for 44 experts and 44 novices many times over, effectively simulating the experiment 100 times. However, in each section we also modify a parameter by either gradually varying the means, varying the number of trials, or removing or replacing certain values, to see how these changes affect the performance models. Of course, each demonstration rests on the data and distributions from the fingerprint comparison task, but we intentionally chose to base our work on this dataset to ensure our methods have direct relevance to real-world human performance studies in forensics.

## Response bias

*Rachel wants to compare experts and novices on a fingerprint matching task like the one described earlier. She presents 12 pairs of prints that are from the same source, and 12 pairs of prints that are from different sources, to 44 experts and 44 novices. She notices, though, that many experts are very hesitant to say “same,” so on most trials, they say “different.” Novices, on the other hand, don’t seem to have much of a bias in either direction. Do differences in response bias present a problem?*

When introducing the various models of performance in previous sections, we noted that dʹ, Aʹ, and empirical AUC, which are grounded in signal detection theory, are supposed to account for response bias, whereas measures like proportion correct do not. There are many forensic disciplines in which examiners invariably exhibit a response bias. Fingerprint examiners, for instance, tend to err toward saying “different source” more than “same source” (Tangen et al. 2011), whereas firearms examiners appear to be more liberal in their responses (Mattijssen et al., 2020). For the purposes of evaluating human performance in pattern matching, however, response bias must be disentangled from accuracy because response bias can confound accuracy (Smith & Neal, 2021).

**Fig. 5:**
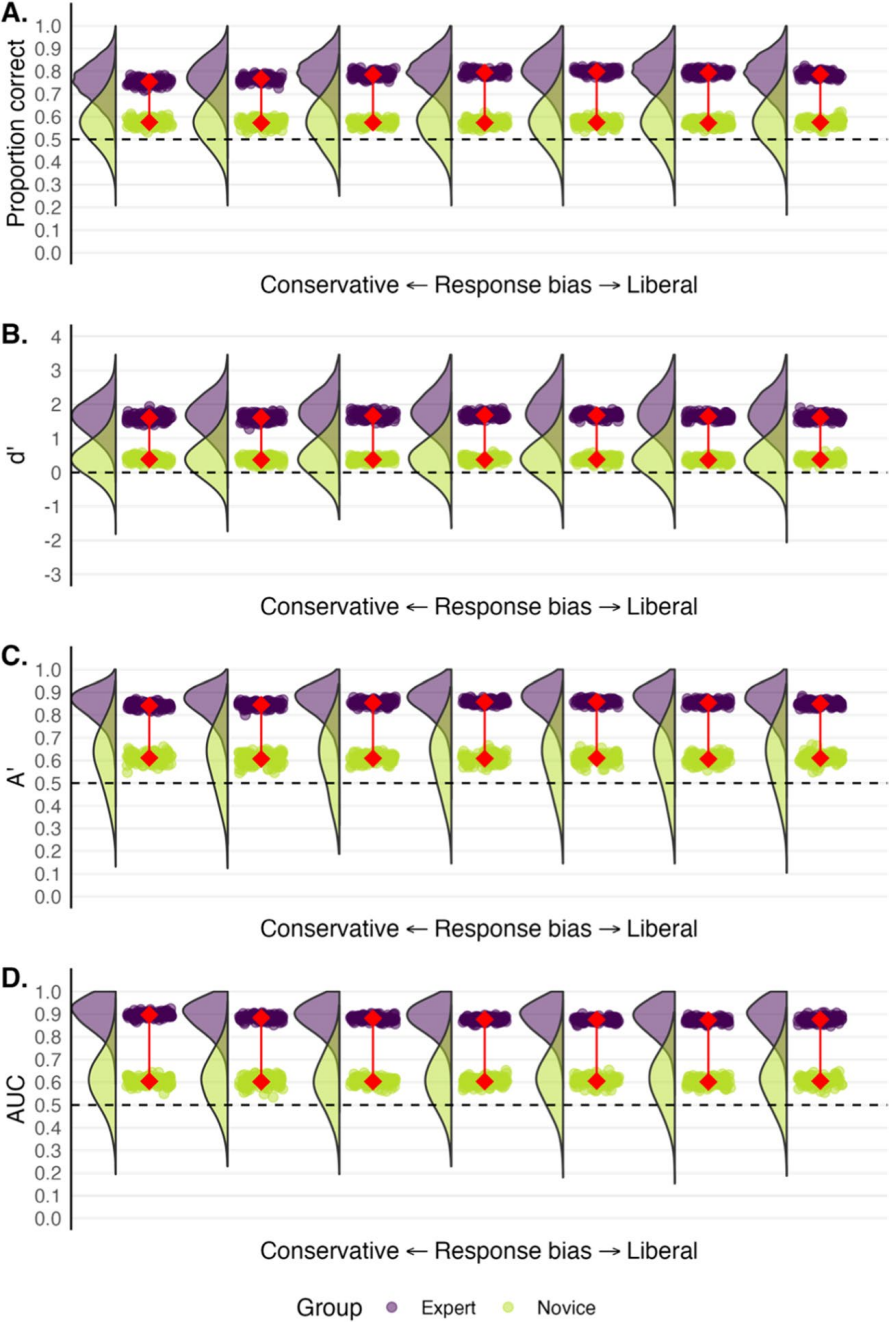
Expert (*purple*) and novice (*green*) performance varying the experts’ response bias. *Rainclouds* depict the distribution of participants’ scores across 100 ‘simulated’ experiments. Each *drop* depicts a group mean. The *connected red points* represent the average of the group means. The *middle plot* depicts real-world data. Plots further to the left depict more conservative responding from experts (less willing to say “same-source”). Plots further to the right depict more liberal responding from experts (more willing to say “same-source”). Novices have the same response bias in each plot. *Dashed lines* represent chance performance

To what extent does a more liberal response bias (saying “same” more), and a more conservative response bias (saying “different” more), affect estimates of performance? We put this claim to the test by gradually either increasing or decreasing the response bias of the expert participants. We gradually varied their mean confidence ratings for the same-source and different-source cases (see Fig. 5). The middle plot depicts expert and novice performance with values corresponding to the actual experiment. The plots further to the left illustrate expert performance as their responses become more conservative; for each successive plot, the mean confidence rating for the same-source and different-source trials both decrease by .5 on the 12-point scale. The plots further to the right illustrate the change in performance as responses become more liberal; the mean confidence ratings for both same-source and different-source trials increase by .5 for each successive plot.

Figure 5 shows that experts consistently outperform novices across all performance models despite changes to their response bias. However, the model that one uses can influence the extent of the expert-novice difference somewhat. For example, the expert-novice differences as measured by Aʹ and dʹ appear least affected by changes to response bias. Proportion correct, on the other hand, appears to slightly underestimate the expert-novice difference as the experts’ response bias becomes more pronounced. Empirical AUC for experts also appears to increase slightly as responding becomes more conservative.

### Summary and considerations

Our data indicate that the chosen model had little effect on performance despite shifts in the experts’ response bias. That said, dʹ and Aʹ appeared slightly more robust than proportion correct and empirical AUC to these shifts. There are, however, caveats to these results. The experts in our original dataset were generally quite confident in their decisions on our 12-point scale, meaning that the underlying signal and noise distributions for our data were not precisely what some theoretical models might assume. Experts also significantly outperformed novices on the task, and while this is not surprising in expert-novice research, a large performance gap can mean that small changes in either group's performance appears less striking. Relatedly, the shifts we made to the experts’ response bias may not have been extreme enough to greatly affect performance estimates. Although not obvious in our case, signal detection models (like dʹ, Aʹ and empirical AUC) generally have more utility than summary metrics like proportion correct when dealing with extreme response bias. We explain why this is so in the introduction. Moreover, the problem of conflating response bias and accuracy can be compounded by differences in prevalence. We turn our attention towards such a scenario next.

## Prevalence rates

*Sam intends to compare fingerprint matching experts and novices. However, he only has a limited number of fingerprints from different sources. He decides to present participants with more trials from the same source than from different sources. Sam also expects that experts will reply more conservatively than novices (saying “different” more frequently than “same”), whereas novices will be more liberal in what they consider to be from the same source. When comparing experts and novices, does an unequal proportion of same-source and different-source trials pose a problem?*

The effect of response bias can be exacerbated when the ratio of same-source and different-source trials is unequal. Growns and Kukucka (2021) have shown that when the proportion of same-source trials is low, the proportion of misses increases. However, when the proportion of same-source trials is high, the proportion of false alarms increases. Imagine a situation in which 90% of trials come from the same source and only 10% come from different sources. Saying “same” on every trial (i.e., an extremely liberal response bias) would allow a person with no knowledge or expertise with fingerprints to be 90% correct in their decisions. In contrast, a competent examiner who responds somewhat conservatively – to avoid false alarms – may have a smaller proportion correct than this novice merely because the majority of print pairs originated from the same source.

In the real world, the prevalence of signal to noise may vary significantly. In baggage screening, for instance, potentially dangerous items appear in only a fraction of cases (Van Wert et al., 2009; Wolfe et al., 2005, 2007). Several forensic studies (e.g., Growns & Kukucka, 2021; Growns et al., 2022; Papesh et al., 2018; Weatherford et al., 2021) have demonstrated that performance can vary significantly based on the proportion of same-source to different-source trials. In fields like fingerprint identification and forensic face matching, the ground truth proportion of same-source versus different-source cases cannot be known for certain. However, an unequal number of signal trials and noise trials will affect measures of performance because sensitivity and specificity are given different weighting.

**Fig. 6:**
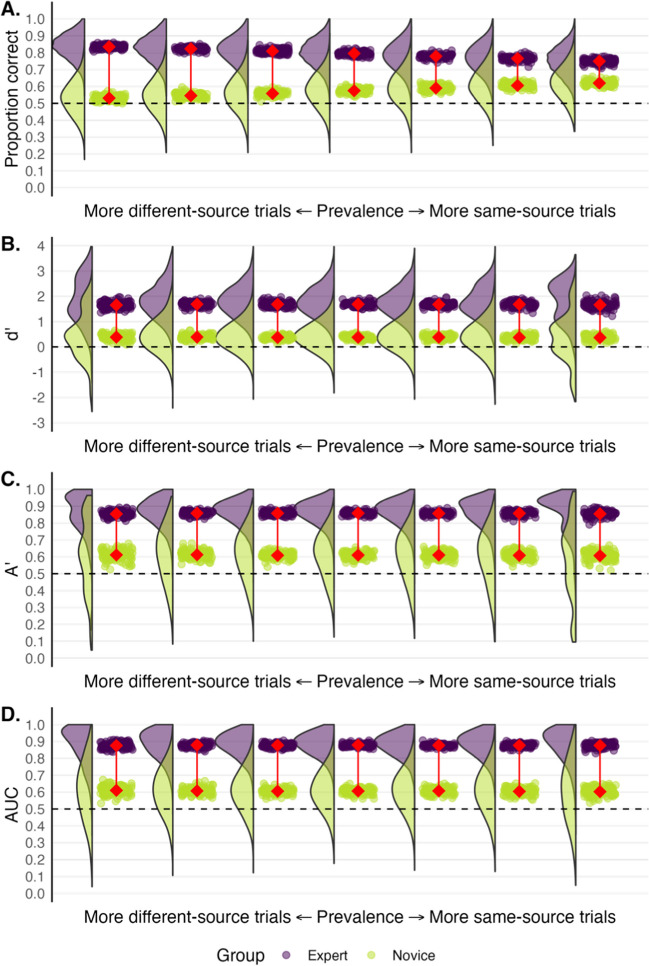
Expert (*purple*) and novice (*green*) performance varying the prevalence of same-source and different-source cases. *Rainclouds* depict the distributions of participants’ scores on 24 trials across 100 ‘simulated’ experiments. Each *raindrop* depicts a group mean. The *connected red points* represent the average of the group means. The *middle plot* depicts performance with 12 same-source and 12 different-source cases. Plots further to the left show performance when there are relatively more different-source prints. Plots further to the right show performance when there are relatively more same-source prints. *Dashed lines* represent chance performance

We resampled data from distributions based on the means and standard deviations for confidence in the fingerprint matching experiment described earlier. Note that the experts had a somewhat conservative response bias in this task whereas novices were more liberal. For each hypothetical participant, confidence ratings for 24 trials were sampled. However, we either increased or decreased the number of same-source trials relative to different-source trials. We present the results in Fig. 6. The middle plot depicts performance when the number of same-source and different-source trials was equal (12 of each). As the plots move to the left, the number of trials from the same source decreases by three, and increases by three moving right. The leftmost plot depicts performance when just three of the 24 trials (12.5%) were from the same source, whereas the rightmost plot depicts performance when 21 of the 24 trials (87.5%) were from the same source.

Figure 6 shows that for proportion correct, experts performed much closer to novice levels when most trials came from the same source. Conversely, the difference between experts and novices increased when there were more different-source trials. Changing the ratio of trials had relatively little effect on dʹ, Aʹ and empirical AUC, but the group means at extreme ends became more variable.

### Summary and considerations

The proportion of same-source versus different-source trials in an experiment can have a significant impact on an individual’s or a group’s performance when using proportion correct. To eliminate the confound that response bias may have on performance, researchers could ensure that the proportion of same-source and different-source trials is roughly equal. Relative to proportion correct, signal detection models such as dʹ, Aʹ or empirical AUC are also less affected when there is an unequal proportion of same-source versus different-source trials.

## Inconclusive responses

*Chloe wishes to compare experts and novices on a fingerprint matching task. She offers three response options to participants: same-source, different-source, and inconclusive. A response that is inconclusive would suggest that based on the information provided, the participant cannot determine whether the prints match. Because there are three response options, Chloe finds it challenging to apply signal detection theory to her data and is concerned that the different inconclusive response rates may present confusion.*

In forensic disciplines such as firearms, shoe marks, handwriting, and fingerprints, it is typical for examiners to reach an inconclusive judgment. When an examiner cannot determine whether evidence originated from the same source as a reference sample, the evidence is deemed *inconclusive*. The option to respond “inconclusive” allows examiners to refrain from making a determination and mitigates the possibility of a false identification or false exclusion (Arkes & Koehler, 2022). So far, we have discussed scenarios where examiners only have two options: same source or different source. What happens when a third option is introduced? We can take the expert data from the prior fingerprint matching experiment and classify all ratings of six and seven (ratings of least confidence) as “inconclusive” to determine how inconclusive judgments could impact diagnostic accuracy (see Table 3).

**Table 3:** Classification table for expert participants (all ratings of 6 or 7 are coded as inconclusive responses)

Expert says						
		“Same”	“Different”	“Inconclusive”	Row total	
Ground truth	Same source (targets)	359 (Hits)	87 (Misses)	69	515	Sensitivity = 69.71% (359/515)
	Different source (distractors)	43 (False alarms)	420 (Correct rejections)	78	541	Specificity 77.63% (420/541)
	Column total	402	507	147	1056	
		PPV = 89.30% (359/402)	NPV = 82.84% (420/507)	IPPV = 46.94% (69/147), INPV = 53.06%		

Comparing Tables 1 and 3 reveals that the addition of an inconclusive response option boosts both the PPV and NPV. This makes sense given that the more challenging trials in which examiners were less confident have been removed from the calculation. As indicated previously, these predictive values may be of most relevance to factfinders since they convey a sense of how certain one should be about a conclusion. Permitting examiners to respond with “inconclusive” may be advantageous in court settings since it appears to increase confidence that a determination (if conclusive) will be in line with ground truth. However, this trade-off is offset by the fact that some cases that would have been correctly judged to be from the same source or from different sources are now classified as inconclusive.

The inconclusive positive predictive value (IPPV) in Table 3 suggests that when an examiner says “inconclusive,” the chance that the two impressions came from the same source is 47%, which means there is a 53% chance that the fingerprints came from different sources (inconclusive negative predictive value; INPV). Factfinders might therefore be less confident in an instance where an examiner says “inconclusive.” When a researcher has data on how many trials an examiner responds with “inconclusive”, then we recommend that this be taken into account when measuring PPV and NPV because these data are relevant. If inconclusive was a response option and not chosen, then a conclusive same-source or different-source decision should be more convincing.

The values for sensitivity and specificity in Table 3 are lower than in Table 1 because the inconclusive responses (in Table 3) are added to the tally in the denominator but not the numerator when calculating each performance estimate. That is, an inconclusive decision reflects neither a hit (numerator for calculating sensitivity) nor a correct rejection (numerator for calculating specificity), but they are nonetheless a decision to be counted in the base rates for each true state. There is currently some debate over whether or not inconclusive responses should be counted as errors (see Arkes & Koehler, 2022; Biedermann & Kostoglou, 2021; Dror & Scurich, 2020; Morrison, 2022). Regardless of their classification, inconclusive decisions are decisions nonetheless, and a full consideration of examiners’ performance ought to take them into account. Researchers interested in validating a decision-making system, forensic methodology, or new processes in a particular forensic laboratory, for example, may be concerned by these changes to sensitivity and specificity because they suggest that including an inconclusive response option can artificially inflate or reduce error rates. Where inconclusive responses have been allowed, one solution to quantifying performance is simply to subdivide the remaining outcomes. Using Table 3, of the same-source trials that were not identified, 56% were misses and 44% were inconclusive. Of the different-source trials that were not excluded, 36% were false positives and 64% were inconclusive. However, this solution does not make it easy to compare performance between examiners, groups, or techniques.

Consider that by responding “inconclusive” to every case they see; examiners can avoid making any mistakes at all (in the sense of true misses and false alarms). Though this example is extreme, we can turn to a real-world study by Bird and colleagues (2010) in which professional handwriting examiners were compared to novices in their ability to distinguish between genuine and disguised handwriting samples. In this situation, it would be important to determine how effectively each group can distinguish genuine samples (signal) from disguised samples (noise). In each case, however, participants were given three response options: identify (same source), exclude (different source), or inconclusive (unable to identify or exclude). Professional examiners were correct on 73% of the trials and responded “inconclusive” on 23% of the trials. Novices were correct on 80% of trials and responded “inconclusive” on 8% of trials. The number of correct responses by novices was higher than that of examiners; did novices therefore do better than examiners? Or did examiners do better because they made errors only 4% of the time, whereas novices made errors 12% of the time?

**Fig. 7:**
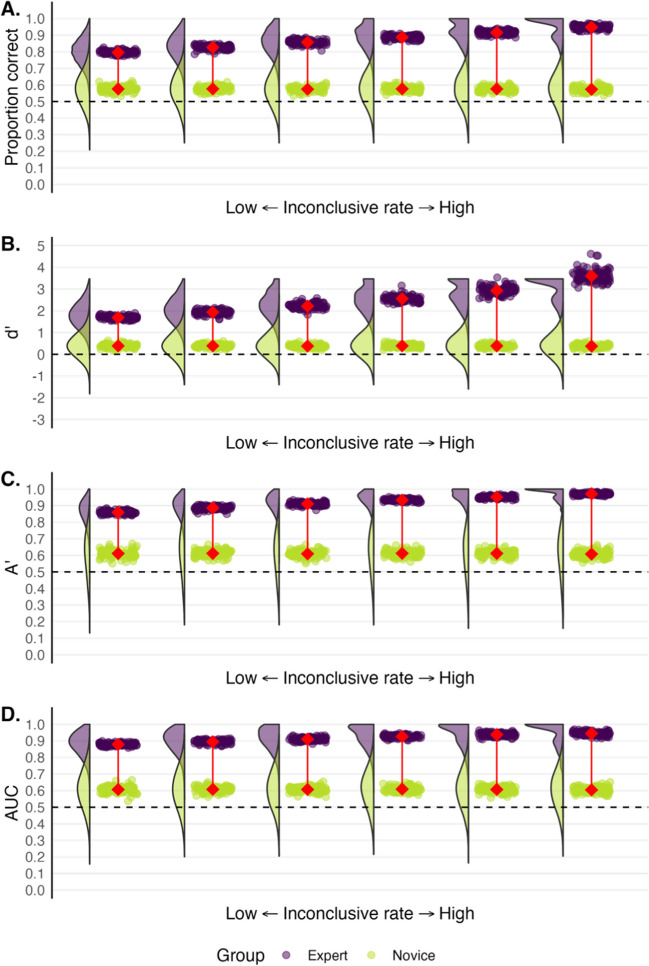
Expert (*purple*) and novice (*green*) performance as if experts become progressively more likely to say “inconclusive.” *Rainclouds* depict the distributions of participants’ scores across 100 ‘simulated’ experiments where each *drop* depicts a group mean. The *connected red points* represent the average of the group means. In the plot on the far left, experts never respond with “inconclusive”. As the plots move right, experts respond “inconclusive” more often: the interval of confidence ratings coded as “inconclusive” increases by two points (on a 12-point scale) for each successive plot. Trials rated as “inconclusive” were excluded from performance calculations. *Dashed lines* represent chance performance

Based on the above findings, Bird and colleagues drew the conclusion that handwriting expertise requires the ability to determine when there is sufficient information to make a determination. However, a researcher could simply instruct novices to make a conclusive decision only when they are extremely confident, which would likely increase the number of inconclusive decisions. If judging sufficiency were an ability, it ought to depend much less on the characteristics or instructions of the task. This behavior might be better characterized as a willingness or bias to say ‘inconclusive’. The performance of a confident participant, who is more likely to make a call, and a cautious participant, who is less likely to make a call, depends a lot on how inconclusive decisions are factored into the overall evaluation of performance. We explored how inconclusive responses affect performance estimates (see Fig. 7). Once again, we used real expert and novice fingerprint matching data, but gradually increased the experts’ propensity to respond with “inconclusive”.

The data underlying the leftmost plot in Fig. 7 offers a baseline where none of the examiner or novice responses were replaced with “inconclusive.” However, the plots further to the right depict performance as more ratings are substituted for “inconclusive.” All confidence values between 5.5 and 7.5 were coded as inconclusive for the plots second from the left. All ratings between 4.5 and 8.5 were coded as inconclusive for the plots third from the left, and so on. Importantly, trials where experts responded with “inconclusive” were not included in computing performance.

Figure 7 illustrates that when inconclusive trials are eliminated from the calculation of performance models, performance appears to improve. Trials judged to be inconclusive are challenging by definition, so removing them makes performance look better. When participants are able to provide inconclusive responses, it would therefore be misleading to use signal detection models, as any value would be affected by how willing a participant is to make a call. Signal detection theory relies on a binary outcome, but this is no longer true when inconclusive decisions are allowed. Moreover, a judgment of similarity (e.g., same-source vs. different-source) is distinct from a judgment of whether sufficient evidence exists to make a call (conclusive evidence vs. inconclusive evidence).

**Fig. 8:**
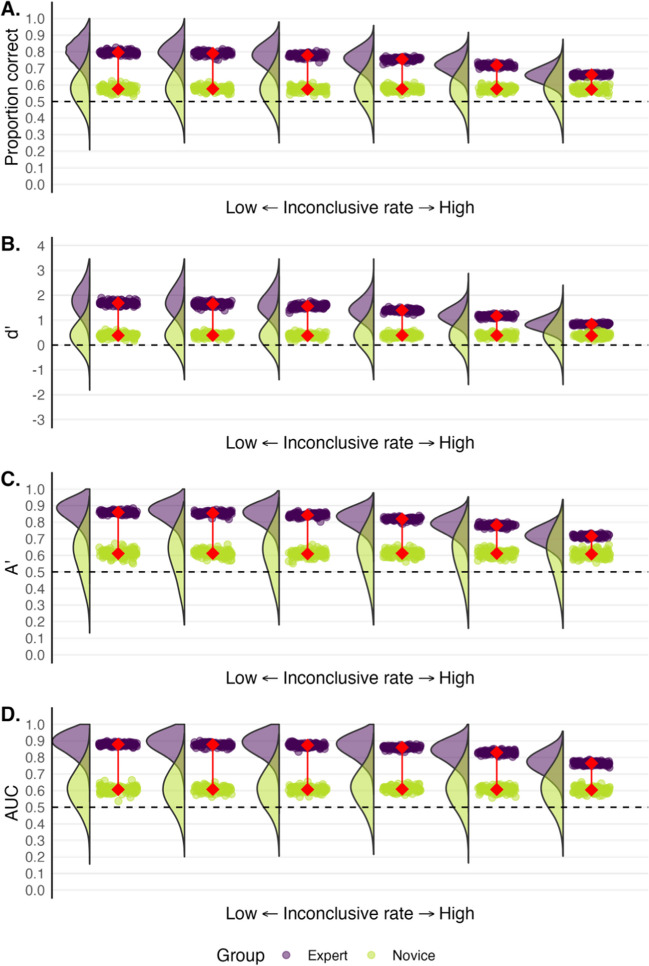
Re-presentation of expert (*purple*) and novice (*green*) data from Fig. 7 as if experts are gradually more willing to say “inconclusive,” but with inconclusive responses now coded as half correct and half incorrect. *Rainclouds* depict the distributions of participants’ scores across 100 ‘simulated’ experiments with each *drop* depicting a group mean. The *connected red points* represent the average of the group means. The plot on the far left depicts performance when experts never respond with “inconclusive”. Plots moving right depict performance as if experts select “inconclusive” more often. *Dashed lines* represent chance performance

Even without knowing what someone would have decided if they had not said “inconclusive,” it could be assumed that when someone says “inconclusive,” they are truly undecided about whether the evidence comes from the same source or a different source. Given this assumption, we can now include all trials, including inconclusive decisions, when calculating performance. In Fig. 8, we display the same data as Fig. 7, but all inconclusive responses have now been substituted for a confidence rating of 6.5 (the midpoint between 1 and 12) when calculating empirical AUC, and a score of 0.5 for accuracy (the midpoint between 0 and 1). None of the novice responses were substituted with “inconclusive” as these serve as a baseline. Moving further right in the Fig. 8, an increasingly wider range of expert responses were re-labeled as "inconclusive."

For all models depicted in Fig. 8, expert performance now decreases as the proportion of inconclusive responses increases, approaching novice levels with each consecutive plot. Whereas removing all inconclusive trials can inflate performance, treating an inconclusive response as a coin toss in the mind of the observer reduces performance. An examiner may not be completely on the fence when they say “inconclusive” so adding randomness when a person is better than chance can only harm performance. When there is a choice of whether to include or exclude evidence, an inconclusive response does not necessarily mean that an examiner is completely unsure. Rather, it indicates that they have low confidence in making an accurate decision and may be taking precautions to avoid false positives and misses.

### Summary and considerations

Including an inconclusive response option when measuring performance is problematic because performance can be artificially inflated or reduced depending on how the inconclusive responses are handled. Deciding whether two impressions came from the same source is different from deciding whether there is sufficient evidence to make such a decision. The latter judgment is about the preponderance of evidence whereas the former is about source. Researchers might therefore want to collect a forced-choice response (either on a dichotomous scale or a continuous scale) about source (e.g., same source/different source, identification/exclusion) separately from a response about whether there is sufficient evidence. Inconclusive responses can, for example, be collected before or after the source question using either a two-response question (i.e., conclusive or inconclusive) or a three-response question that reflects case work (i.e., exclusion, identification, inconclusive). Including both questions allows one to learn how many cases (and which ones) evoke an inconclusive response and therefore the ability to compute predictive values that are useful to factfinders, but performance can also then be measured using signal detection theory in a way that is not undermined by the presence of a third response option.

## Task difficulty

*Matt wants to see how well a group of examiners performs compared to novices on a fingerprint matching test with 24 cases, half of which are from the same source and half are from different sources. The expert group is correct in virtually every instance, and he’s impressed by their performance. But then Matt gives the same task to a group of undergraduate students, and he finds that they also do extremely well. Matt is now unsure whether the experts are actually better than the novices, or whether his test is a poor assessment of their abilities.*

Forensic proficiency tests have come under fire in recent years for being too simple. For instance, professional examiners performed exceptionally well on proficiency tests created by collaborative testing systems (CTS), but it was later found that even people with no formal experience with fingerprints could identify many of the test cases correctly (Smith, 2019). Many forensic proficiency tests are too easy (Koehler, 2017). To be considered an expert in a field, one must be able to perform better than untrained individuals in situations where the expert claims to be proficient. When experts and novices achieve similar results on a test, either the experts are not true experts, or the task is not a good indicator of competence. How well a test can distinguish between experts and novices is also largely dependent on the test’s difficulty.

**Fig. 9:**
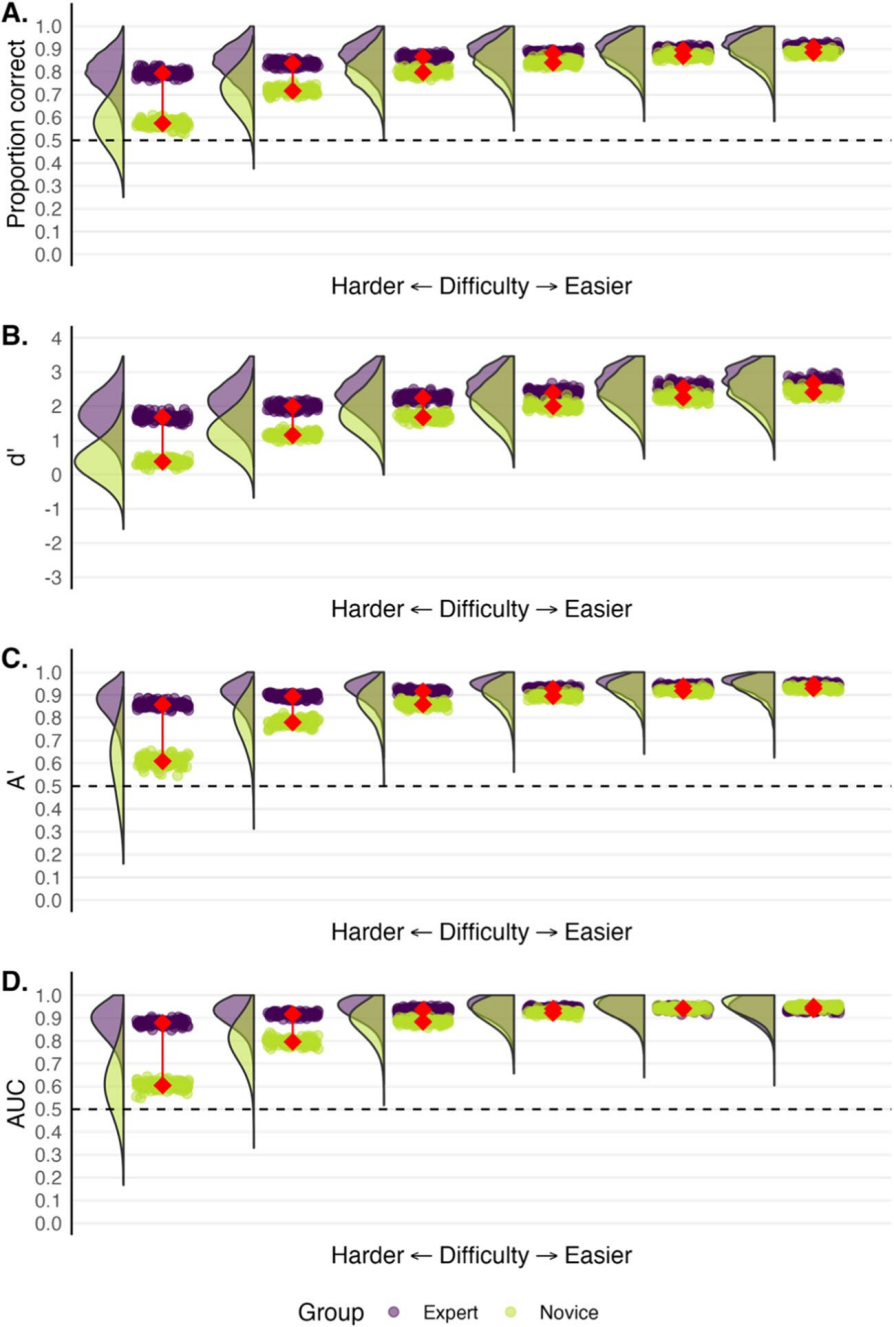
Expert (*purple*) and novice (*green*) performance varying the difficulty of the cases. *Rainclouds* depict the distributions of participants’ scores across 100 ‘simulated’ experiments with each drop depicting a group mean. The *connected red points* represent the average of the group means. The leftmost plot depicts real-world fingerprint comparison performance. The plots further to the right depict performance as if cases become 25% easier. Eventually, the performance of the two groups becomes indistinguishable. *Dashed lines* represent chance performance

We explored how the performance of experts and novices can differ as a test becomes easier (see Fig. 9). We again used the means and standard deviations from the fingerprint task from earlier. Baseline performance is illustrated by the leftmost plots in Fig. 9. Each plot moving to the right shows performance as if the task was made easier. For the same-source trials, the mean for each group was increased to be 25% closer to 12 for each successive plot, and the means for different-source trials were gradually decreased to be 25% closer to 1.

Experts outperformed novices in the real world (leftmost plots in Fig. 9) across all performed models. However, as the trials become easier on average, both experts and novices perform closer to ceiling, and they become virtually indistinguishable across all measurement models. A test’s ability to differentiate between experts and novices is compromised if everyone, experts and novices alike, can achieve a similar level of performance. A similar problem can arise if the test is too difficult, as both experts and novices may struggle to pass it. Because the groups are not as easily distinguishable as they should be, the test fails as a measure of skill.

### Summary and considerations

An expert is someone who consistently outperforms the great majority of other people in a given domain. If a test fails to differentiate between experts and novices, either the experts are not really experts, or the test is a poor indicator of domain expertise. We cannot know which of these propositions is true if a test is given to a group of experts but not to a novice control group. Researchers might consider including a control group when measuring performance in forensic domains, or when validating a test. Likewise, pilot testing the difficulty of test trials helps ensure that a test is neither too easy nor too challenging. Because everyone, including non-experts, will appear to perform well on a simple test, it will be impossible to identify genuine expertise when it exists. A test that is overly difficult will fail to detect true expertise when it exists, as everyone, including experts, will appear to perform poorly. A good test reveals variance in performance.

## Trial sampling

*Brooklyn designs an experiment to see if her training intervention can increase people’s performance on a matching task. She gives each participant in her study the same fixed sequence of 24 trials before training, and then tests them again after training with a completely different fixed sequence of 24 trials. Brooklyn discovers that the group improved from pre- to post-test, but she is uncertain about whether the source of these improvements was the training intervention or because different cases were used at pre- and post-test.*

So far, we have focused on situations in which experts are compared to novices. In contrast, Brooklyn tests a single group of novices both before and after her training intervention. The implications of this section are relevant for any study, but they are especially important when dealing with a small sample size or a limited number of trials. Confounds can be caused by differences in the test cases given to different groups or at different times. In Brooklyn’s case, we don’t know if the trials given before training are harder or easier than the ones given at the end of training. A disproportionate number of easy (or hard) trials in one test, but not another, can skew conclusions about the effectiveness of the training intervention. Maybe the subjects were lucky enough to be tested with more difficult trials first, followed by easy trials after training, or vice versa.

**Fig. 10:**
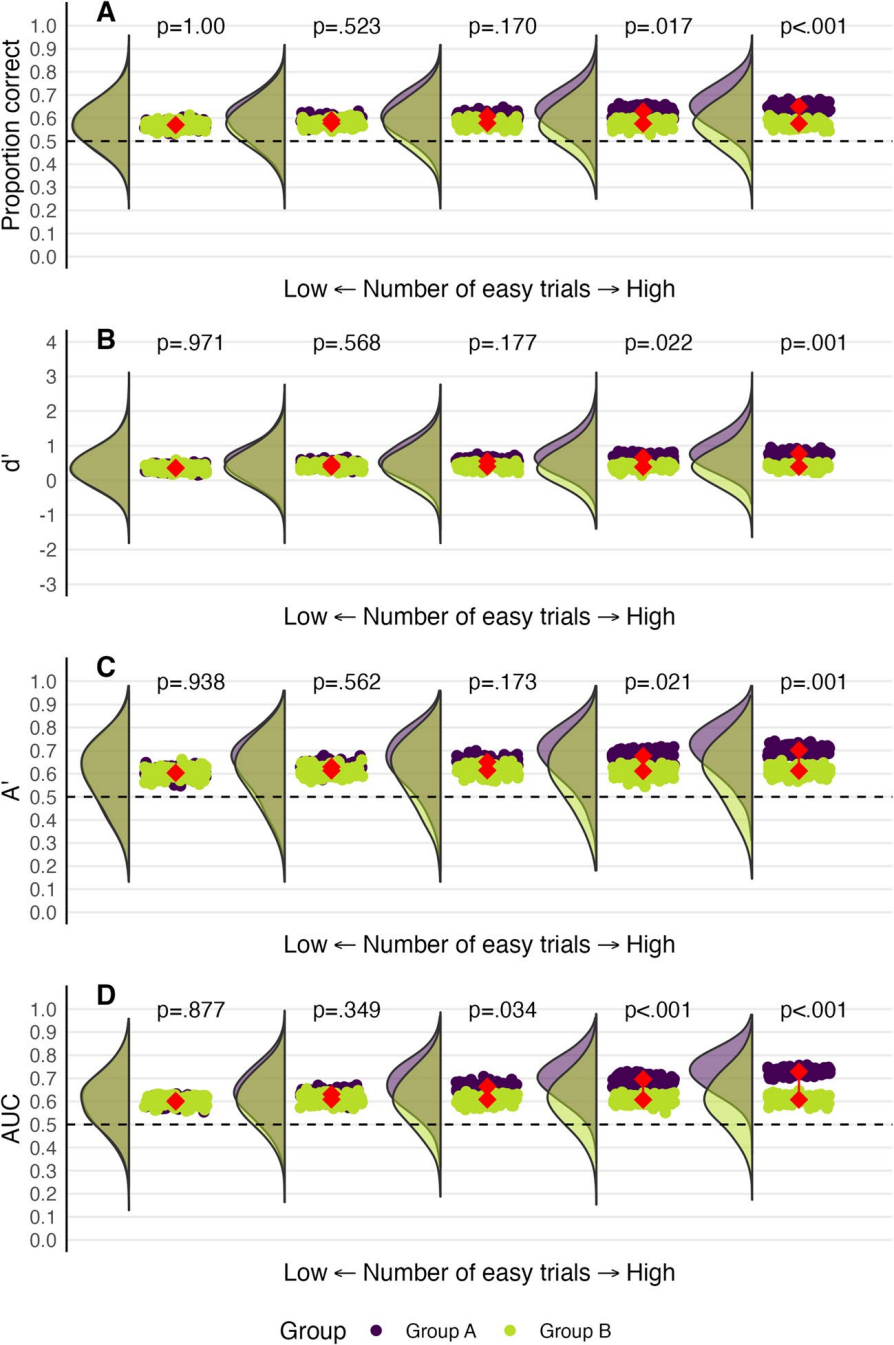
Pre-test novice group (*green*) and a post-test novice group (*purple*) for a training study where the training intervention had no effect on performance. *Rainclouds* depict the distributions of participants’ scores across 100 ‘simulated’ experiments with each *drop* depicting a group mean. The *connected red points* represent the average of the group means. The leftmost plot depicts performance when the ease of the pre-test and post-test were identical. Plots further to the right include an increasing number of easy trials in the post-test case set, from zero on the leftmost plot to four on the rightmost plot. The *p* values displayed correspond to the median *t* test statistic. *Dashed lines* represent chance performance

To demonstrate, we again used the novice means and standard deviations from the earlier fingerprint experiment as the basis for our resampling method. In Fig. 10, novice performance at pre-test is displayed in green, and performance at post-test is displayed in purple. The leftmost plot shows a situation where neither group had particularly easy trials and the two distributions overlap almost entirely. This makes sense as we are assuming that the training intervention had no effect on performance. However, we systematically increased the number of easy trials for the post-test group in each successive plot moving right. Specifically, all participants responded with 1 (“sure different”) on a certain number of non-match trials. We increased the number of easy trials by one for each plot moving right. In the rightmost plot, four of the 24 trials that the post-test group received were easy. The values displayed above each plot indicate the p-value for the median t-test statistic across the 100 resampled experiments.

Figure 10 reveals that even if a training intervention did not improve performance in reality, a small increase of just three easy trials out of 24 (12.5%) at post-test can result in a significant difference in performance between pre-test and post-test more than half the time. In the case of empirical AUC, just two trials were required to produce a consistent significant difference. Using fixed sequences and not providing a control group may result in unintended differences in the cases presented to different groups or conditions. These differences leave the door open for confusion, which limits what researchers can infer about performance.

### Summary and considerations

Fixed sequences can make it difficult for researchers to determine if a difference between two groups, or two timepoints, was caused by an interesting effect or intervention, or whether it was simply due to a difference in the trials presented. There are a variety of solutions to this problem. First, as mentioned in the preceding section, researchers could include a control group in their study design. Even if fixed sequences are used, differences in the trials can be controlled for if participants who receive training are compared to another group that receives no training at all, or another intervention altogether. Researchers can also counterbalance which fixed sequence a participant sees at each timepoint. Some can receive Sequence A at pre-test and Sequence B at post-test, whereas others can receive B first and then A.

Alternatively, one can generate a completely unique, random sequence of trials for each participant, which can be sampled from a larger pool. Though this sampling method may increase noise, it will increase the generalizability of a study’s results because they would be based on a broader cross-section of trials. Researchers could even randomize sequences whilst also ensuring that a member of each group is presented with the same randomized sequence as a member of the other group. This method ensures that results are generalizable while also reducing noise. In fact, in the earlier fingerprint experiment, members in each matched expert-novice pair were shown the same unique, randomized sequence of trials. With all this said, however, fixed sequences can be useful for detecting differences with the greatest sensitivity, particularly in research on individual differences (see Mollon et al., 2017).

## Number of trials

*Jason is aware of Brooklyn's earlier predicament; he knows that the disparities between pre- and post-test performance could be due to a few particularly easy trials. Jason wants to avoid this problem, so he decides to increase the number of trials in his experiment. He suspects that the effect of the easy trials will be negated in the aggregate because these easy trials will have proportionally less impact on overall performance.*

To rule out the possibility that any apparent differences between groups or timepoints are due to chance, researchers may choose to include additional trials in their test. However, participants in high-profile tests like those discussed earlier may only be exposed to fewer than a dozen trials. When evaluating performance, having fewer trials means that performance estimates are less reliable so researchers should be less confident in their conclusions and generalizability of such studies.

**Fig. 11:**
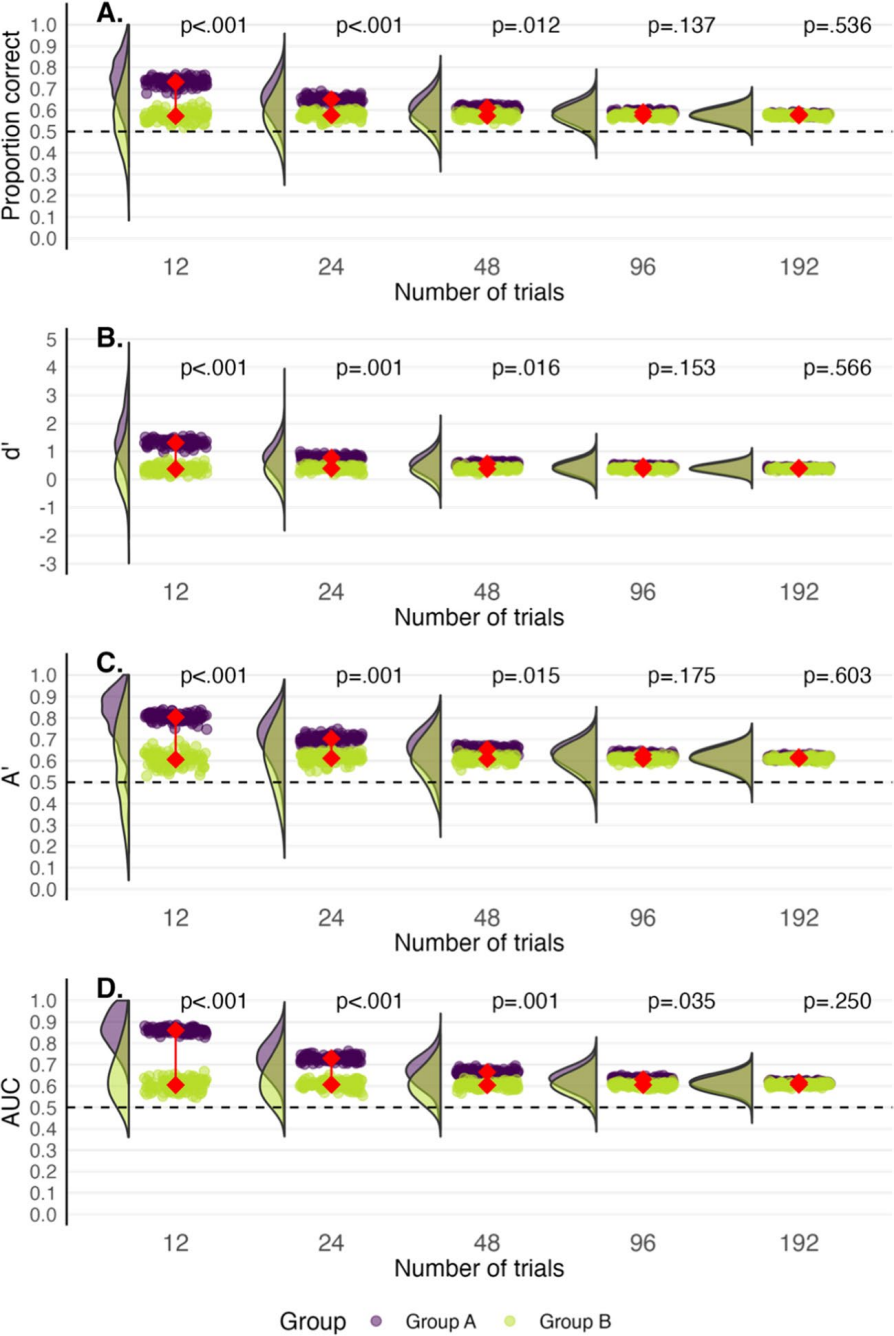
Pre-test novice group (*green*) and a post-test novice group (*purple*) for a training study where the training intervention had no effect on performance, but the post-test case set had four easy trials. *Rainclouds* depict the distributions of participants’ scores across 100 ‘simulated’ experiments and each *drop* depicts a group mean. The *connected red points* represent the average of the group means. In the leftmost plot, 12 trials are presented. The trials double with each successive plot moving right. The *p* values displayed correspond to the median *t* test statistic. *Dashed lines* represent chance performance

We explored how many trials might be required to counteract the influence that a small number of easy trials can have on pre-post performance gains. We used the mean and standard deviation of novice performance in the earlier fingerprint task and assumed that the hypothetical training intervention has no effect on performance. Thus, the groups should not have differed statistically with randomly sampled cases. Four easy trials were sampled at post-test whereas no easy trials were included at pre-test. To mimic the easy trials, we simply ensured that four of the different-source trials received a rating of 1 (“sure different”) from each hypothetical participant. The data are presented in Fig. 11 where the green distributions represent pre-test performance, and the purple distributions represent post-test performance. For each plot moving right, we doubled the total number of trials that we sampled.

Figure 11 shows a lot more variation in performance when there are fewer trials. In addition, the gap between the distributions widens when the total number of trials is small and narrows when the total number of trials increases. Easy trials have less influence on performance when they account for only a small proportion of overall trials. However, up to 96 trials are needed for performance to become non-significant more than half of the time for proportion correct, dʹ and Aʹ. For AUC, up to 192 trials are required. In other words, even a small number of easy trials in one test set and not another can have a major impact on performance, and this issue might not be easily solved by simply adding more trials.

### Summary and considerations

When only a few trials are used to assess performance, the presence of a few easy trials in one group or at one timepoint can significantly affect performance. Increasing the total number of trials can lessen the effect that these trials have, but many trials are required. To circumvent any potential problems posed by specific sequences of trials, researchers may choose to include as many trials as is practical in conjunction with more stringent case selection procedures, such as counterbalancing and random sampling of trials for each participant.

**Table:** 

**Take Home Messages**
**• Determining when and what performance metric to use:** Courts are interested in positive and negative predictive values because they characterize the reliability of an examiner’s judgment. In the hands of a human examiner, however, the general validity of a forensic method is best described by its sensitivity, specificity, and discriminability, which come from signal detection models that take these values into account. **• Transparent research practices:** Specifying design and data analytic decisions prior to analyzing one’s data (preregistration) as well as making materials and data as transparent as possible, will help ensure the reliability of research in forensic science and its practical impact. **• Response bias and prevalence:** Discriminability metrics (dʹ, Aʹ, empirical AUC) are more robust to response bias effects and unequal ratios of same-source and different-source cases compared with metrics that simply count the number of correct vs. incorrect judgments (i.e., proportion correct). **• Inconclusive responses:** The handling of inconclusive judgements can greatly affect estimates of forensic examiners’ performance. Consider collecting inconclusive judgments separately from forced-choice judgments about source. Collecting data for these two claims separately allows one to compute discriminability and response bias irrespective of an inconclusive decision, and to compute PPV and NPV taking into account inconclusive decisions. **• Task difficulty:** Without an appropriate comparison group, it is impossible to determine whether a test was too easy or too difficult for the target expert population (e.g., fingerprint examiners), rendering a single performance estimate for this population worthless. This issue can be resolved by including a comparison group (e.g., non-experts). **• Trial sampling:** Fixed trial sequences, where different participant groups see different sets of cases, can introduce spurious effects in some circumstances. To remedy this issue, consider randomly selecting trials from large case sets for each participant. Other solutions include counterbalancing and yoking participant trial sequences. **• Number of trials:** Experiments with only a small number of trials can produce unreliable performance estimates. Increasing the number of trials is a simple method for improving performance estimation.

## Conclusions

Our goal with this paper was to give useful descriptive guidance for anyone interested in creating and assessing studies that model the performance of forensic examiners and their procedures. We have explained how signal detection theory can be used to conceptualize performance, outlined several commonly used models of performance, and reported new data on the performance of novices and expert fingerprint examiners. We then used these results to define distributions from which we could resample data and explore how different models of performance hold up in a variety of scenarios. In doing so, we offer considerations and solutions to many issues that researchers frequently face when studying forensic examiner performance. Many of these considerations complement advice offered by other scholars such as Martire and Kemp (2018).

Partitioning examiners’ decision outcomes into estimates of positive and negative predictive values, for example, would be most useful in contexts where the probability of the true state (e.g., the prints were made by the same source) given the examiner’s judgment (e.g., when the expert says “same source”) is of primary interest. The PPV and NPV are most likely to be useful when the true state is unknown, like when evaluating the credibility of a forensic examiner’s source-attribution testimony in court. On the other hand, separating examiners’ decisions into estimates of sensitivity and specificity, and/or combining these values using the performance models discussed here, is a better way to answer general questions about the validity of a forensic technique, method, or decision-making system. For these kinds of questions, we need to know the probability of the examiners’ decision (e.g., the expert says “same source”) given the true state (e.g., the prints were made by the same source). If researchers and forensic examiners can agree on how to measure and model performance, make their data available to others, and make their analytic decisions transparent, then it will be possible to gain a better understanding of expert forensic pattern matching ability as well as how to best communicate errors in forensic decision-making to factfinders.

## Data Availability

The de-identified data and analytic scripts can be found on the OSF at https://osf.io/wt2zn/.

## Footnotes

1 Sometimes discriminability is referred to as sensitivity, but we will not use this term because it can be easily confused with another diagnostic accuracy known as sensitivity, which we introduce later.

2 Due to a labelling error, several participants saw 13 distractor trials and 11 target trials instead of 12 of each. Hence, there were slightly more distractor trials overall.
